# Autism Spectrum Disorder in Down Syndrome: Experiences from Caregivers

**DOI:** 10.1007/s10803-022-05758-x

**Published:** 2023-01-09

**Authors:** Noemi Alice Spinazzi, Alyssa Bianca Velasco, Drew James Wodecki, Lina Patel

**Affiliations:** 1 Division of Primary Care, Department of Pediatrics, UCSF Benioff Children’s Hospital Oakland, 5220 Claremont Ave, Oakland, CA USA; 2 Graduate Medical Education, Department of Pediatrics, UCSF Benioff Children’s Hospital Oakland, Oakland, CA USA; 3 School of Medicine, University of California-Davis, Davis, CA USA; 4 Division of Child and Adolescent Mental Health, Department of Psychiatry, Children’s Hospital Colorado and University of Colorado, Aurora, CO USA; 5 Present Address: Lucille Packard Children’s Hospital Stanford, Palo Alto, USA

**Keywords:** Down syndrome, Autism spectrum disorder, Dual diagnosis, Family/caregiver experience

## Abstract

This study aimed to learn about the experiences of families of individuals with a dual diagnosis of Down syndrome (DS) and autism spectrum disorder (ASD) (DS-ASD), and to document the journey from early concerns to diagnosis and intervention. Caregivers completed an online survey describing their journey raising a child with DS-ASD. Survey responses were analyzed qualitatively and coded into categories to highlight common themes. Stereotypy, severe communication impairments, and behavioral difficulties prompted caregivers to pursue further evaluation. There was a mean 4.65-year gap between first noticing symptoms and receiving an ASD diagnosis. Several therapeutic interventions were identified as beneficial, including behavioral and communication support. Caregivers expressed frustration and described high levels of stress and social isolation. The diagnosis of ASD in children with DS is often delayed, and caregivers’ initial concerns are frequently dismissed. Raising a child with DS-ASD can lead to social isolation and elevated caregiver stress. More research is needed to tailor diagnostic algorithms and therapeutic interventions to the unique needs of this patient population. Caregivers yearn for improved understanding of DS-ASD, more targeted therapies and educational programs, and more overall support.

## Introduction

Autism spectrum disorder (ASD) is a common co-occurring condition in individuals with Down syndrome (DS). Estimates of prevalence range broadly from 5 to 39% (Capone et al, 2005; DiGuiseppi et al, 2010; Hepburn et al, 2008; Lowenthal et al, 2007; Moss et al, 2013; Richards et al, 2015; Starr et al., 2005), depending on the assessment procedures and diagnostic criteria used. This suggests that ASD is much more prevalent in children with DS than in the general population, where the estimated prevalence of ASD is 1.85% (Maenner et al., 2020).

Despite this well-described co-occurrence, ASD is often diagnosed at a later age in patients with DS, if they are diagnosed at all (Howlin et al., 2008; Rasmussen et al, 2001). Thus, children with a dual diagnosis of Down syndrome-autism spectrum disorder (DS-ASD) may miss the window for intensive early intervention, which has been shown to lead to better developmental outcomes in individuals with idiopathic ASD (Greenspan, 1994; Lovaas, 1987; Webb et al, 2014). Though some studies have shown that developmental regression associated with ASD occurs later in children with DS compared to children without DS (Castillo et al, 2008; Warner et al, 2014), other factors, such as decreased awareness of DS-ASD, differences in ASD symptomatology, and diagnostic overshadowing, can lead to a later diagnosis. Older literature described people with DS as friendly and affectionate (Gibbs & Thorpe, 1983), and suggested that ASD rarely co-occurs in individuals with DS (Gillberg et al, 1986; Rutter & Hersov, 1985; Rutter & Schopler, 1988). A recent study comparing children with DS-ASD to ASD alone demonstrated that though these two groups have similar profiles of communication and repetitive behaviors, children with DS-ASD have milder social difficulties, more imitative play, and less impairments in eye gaze and social smiling (Warner et al., 2017). Further, recognizing ASD in children with DS is a challenge due to overlapping symptomatology between intellectual disability and ASD, as both can be associated with deficits in communication, play, and behavioral difficulties. ASD-like symptoms are also more frequently seen in individuals with DS without a co-morbid ASD diagnosis than in the neurotypical population (Channell et al, 2015), which may lead providers to believe that their patient’s neurocognitive profile is solely attributable to intellectual disability, rather than suspecting an additional diagnosis of ASD (Howlin et al, 2008; Reiss et al., 1982).

Parent perspectives provide valuable input in developing tailored care models for different patient populations (Findlen et al., 2019). Parent observations, including developmental and behavioral concerns, have been shown to be predictive of a subsequent diagnosis of ASD (Sacrey et al., 2015). While studies have inquired about the experience of caregivers of children with DS or ASD (Ashworth et al., 2019; Dabrowska & Pisula, 2010; King et al, 2012; Siklos & Kerns, 2006), little is known about the perspective of caregivers of individuals with DS-ASD. The goal of this study was to learn about caregivers’ perspectives, observations, and lived experience with their child with DS-ASD, and to document the journey from early concerns to diagnosis and intervention. Clinical experience informed the hypothesis that caregivers encountered barriers to obtaining an ASD diagnosis, and that their parenting experience was different from that of caregivers of individuals with DS alone.

## Methods

### Survey Development and Data Collection

A survey was developed by the ASD workgroup of the Down Syndrome Medical Interest Group—USA (DSMIG-USA) (Supplemental Information 1). The goal of the survey was to better understand the experiences of families with children with known or suspected dual diagnosis of DS-ASD. The study team developed 17 open-ended questions, including 6 multistep questions, to assess participants’ experiences with their child in the early years prior to diagnosis of ASD; interactions with the medical and education systems in obtaining a diagnosis of ASD; perspectives on what interventions and supports have been helpful; and reflections on how the dual diagnosis of DS-ASD has affected their family unit. The survey specifically inquired about the age at which the individual had been diagnosed with ASD, what type of professional made the diagnosis, and how the diagnosis was obtained (i.e. clinical diagnosis, questionnaires, caregiver interview, developmental testing, psychoeducational testing). The only demographics solicited were age of the individual with DS-ASD at the time the survey was completed, and city and state where the respondent resided. The study was approved by an Institutional Review Board.

All survey respondents were members of the DS-ASD Connection, a closed online community for families who have a child with a confirmed or suspected dual diagnosis of DS and ASD. The survey link was posted on the organization’s closed Facebook page and distributed to members in the DS-ASD Connection’s e-newsletter. The survey was available in English and was open from May 5, 2019 to June 15, 2019. A reminder notification was sent four weeks after the initial survey distribution, and the enrollment period was closed two weeks after the last survey submission. To maintain anonymity, the survey had to be completed in a single session; partially completed surveys could be submitted.

### Data Analysis

Surveys were excluded from analysis if the responses to questions related to the diagnostic process indicated that the individual with DS had not actually received a formal diagnosis of ASD, or if the caregiver only answered demographic questions. For the remaining surveys, each question was analyzed independently, regardless of whether the respondent skipped other questions in the survey. This was done to maximize the sample size for each independent question and resulted in different sample sizes for each question.

Initial aggregation, cleaning, and sorting of the data was done on Microsoft Excel. Qualitative data analysis was informed by thematic analysis methodology (Creswell, 2014), with a coding scheme that was developed by the study team through several consensus discussions that included clinicians experienced in caring for children with DS, ASD, and DS-ASD. General themes were identified for each survey item that required qualitative interpretation of parent responses. Multiple clinicians independently coded the data, and interrater concordance kappa statistic (Fleiss, Levin & Paik, 2003) was 0.92; all divergences in coding were reviewed as a group with consensus reached after reviewing each case. Age-related questions were analyzed quantitatively. A simple linear regression was performed on age variables and displayed as a line plot with an adjusted R2 correlation coefficient. All analytical computing including graph, table, and statistical parameter formation was completed on RStudio and RMarkdown.

## Results

### Demographics

The survey was distributed in the DS-ASD Connection Facebook group, which has a total of 486 members. The survey reached 172 caregivers (35% of members), who engaged with the survey link. Of those 172 caregivers, the response rate was 39% (n = 67). Nine surveys were excluded as they only contained demographic information, and 11 were excluded because responses indicated that the child was never formally diagnosed with ASD. The remaining 47 surveys were included in the analysis. Most survey participants were based in the United States. The median age of the respondent’s child with DS-ASD was 17.00 years old (SD = 8.37) at the time of survey completion, and 70% (n = 33) of them were male. 

### Early Years and Diagnosis

#### Age of Diagnosis

**Fig. 1:**
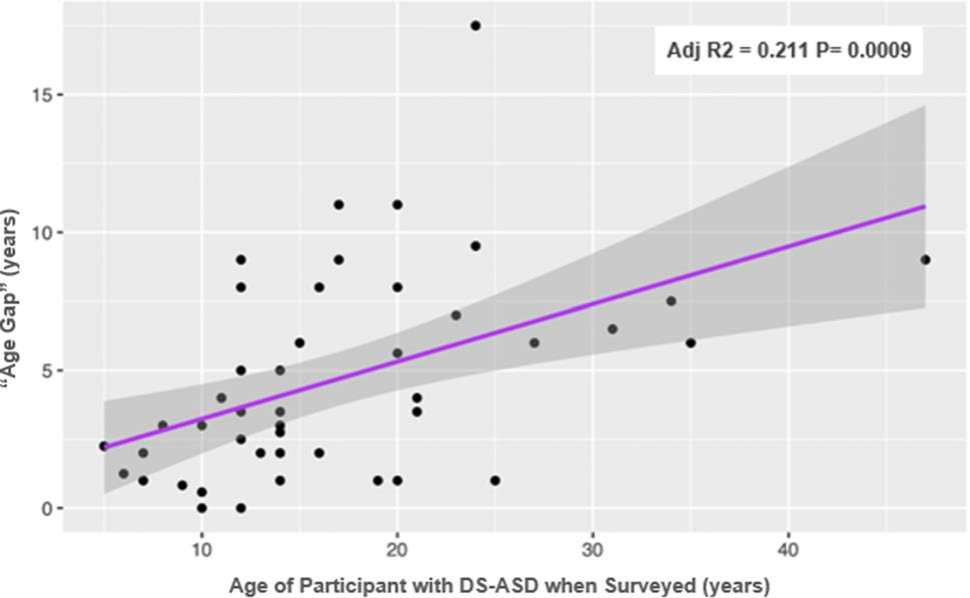
Age Gap vs. Age of Participant with Down syndrome – Autism Spectrum Disorder (DS-ASD) when surveyed

In this survey, caregivers reported first noticing differences in their child compared to other children with DS at a mean age of 3.87 years old (SD = 2.56). However, most children did not receive a diagnosis of ASD until school-age, at an average age of 8.52 years old (SD = 4.77). There was a mean delay of 4.65 years (SD = 3.67) between when symptoms were first noticed by parents and when the child received an ASD diagnosis (Table 1). This “age gap”, defined as difference between reported age when parents noticed signs of ASD and reported age when a formal diagnosis was received, was found to be positively correlated with the age of the child at time of survey participation (Adjusted R2 = 0.211, P = 0.0009) (Fig. 1).

**Table 1:** Age of Child at the time of initial concern for divergent development and diagnosis of Autism Spectrum Disorder (ASD) (n = 45)

Category	Mean	Median	Range	SD
Age at initial concern for divergent development (years)	3.87	3.00	0.25–12.00	2.56
Age at diagnosis of ASD (years)	8.52	7.00	1.50–21.00	4.77
Number of years between first concern and diagnosis (“Age Gap”)	4.65	3.50	0.00–17.50	3.67

#### Initial Symptoms

The survey inquired about symptoms that prompted caregivers to suspect that their child was different from other children with DS. The most commonly reported initial concerns were stereotypic behaviors (n = 24, 53%), which included repetitive behaviors (e.g. hand flapping, stimming, head banging), perseverative behavior (e.g. staring at ceiling fans or lights), and limited interests (e.g. playing with same toy over and over). Lack of interest in social relationships and poor shared attention (n = 22, 49%), as well as communication deficits (n = 18, 40%), were also frequently observed in the early years. Caregivers also reported noticing sensory sensitivity or defensiveness (n = 13, 29%); poor eye contact (n = 12, 27%); mood or behavior disturbances including tantrums, hyperactivity, anxiety (n = 11, 24%); and abnormal patterns of play (n = 10, 22%), including preferring to playing alone, lack of imaginative play and inappropriate use of toys. Many respondents reported slower progress in developmental milestones (n = 12, 27%) compared to other children with DS, and a subset mentioned developmental regression (n = 8, 18%) as the presenting concern.

#### Reporting of Concerns

Most participants expressed their initial concerns to the child’s primary care provider (PCP) (n = 22, 47%) or professionals in the education/therapeutic fields (n = 27, 57%) such as teachers, speech/ physical/ occupational therapists, and early intervention specialists. A smaller subset (n = 12, 26%) initially expressed concern to a medical specialist, such as a developmental and behavioral pediatrician, neurologist, geneticist, or DS specialist (providers who work in DS clinics or centers, or otherwise see a large population of patients with DS). Of those who expressed their initial concerns to their primary care provider, 82% (n = 18) felt that their provider did not have the knowledge or experience with DS-ASD to further guide them (Table 2).

**Table 2:** Who caregivers reported concerns to, and whether they were perceived to have the knowledge to guide further evaluation (n = 47)

Recipient of concern	Family (n = 6)	Educator/Therapy provider (n = 27)	Pediatrician/Primary care provider (n = 22)	Medical specialist (n = 12)
Knowledgeable	0 (0%)	6 (22%)	4 (18%)	10 (83%)
Not knowledgeable	6 (100%)	21 (78%)	18 (82%)	3 (25%)

### Supports, Services, and Interventions

Parents were surveyed on their child’s relative strengths and difficulties. They were also asked to reflect on the supports and interventions that were helpful for their child. All survey respondents were able to identify at least one strength in their child, but the responses were too diverse to categorize. Some examples of parent-reported strengths in their child included affection, compassion, and perseverance. The most frequently reported challenges were communication deficits (n = 25, 57%), cognitive rigidity (n = 18, 41%), deficient social skills (n = 13, 30%), and/or mood and behavioral issues (n = 11, 25%). 

When asked about interventions that had been helpful for their child, 37% (n = 16) of respondents mentioned Applied Behavioral Analysis (ABA) therapy; 35% (n = 15) listed therapeutic services (including speech, physical, and occupational therapy); and 14% (n = 6) referenced specialized DS or autism centers. Respondents were divided when asked whether school was a helpful resource—51% (n = 20) felt that school met their child’s needs, 36% (n = 14) did not feel that school was helpful, and 13% (n = 5) felt school was helpful in some respects but not in others.

### Family Impact

The main source of frustration reported by participants was providers’ lack of knowledge around DS-ASD (n = 11, 25%). Many caregivers endorsed difficulties communicating with their child and managing behaviors, and several respondents felt that their child’s therapeutic services were inadequate, as they were not appropriately tailored.

When asked to identify the greatest impact that having a child with DS-ASD has had on the family unit, 38% (n = 16) of participants named feeling isolated. Many reported that caring for their child was exceedingly time consuming and required a career change. Despite the challenges of raising a child with DS-ASD, 38% (n = 16) of parents shared that the experience has made them more empathetic, stronger, and more grateful. Many respondents mentioned feeling a like they did not belong within either the DS or the ASD communities. Themes from survey responses, including illustrative quotes from this section of the survey, are shared in Table 3.

**Table 3:** Themes and Quotes from the “Family Impact” Section of the Survey

Topic	Theme	Illustrative quote(s)
Challenges and frustration	Communication impairment	*“The lack of communication from our son. He can't express his needs/ wants/feelings.”*
	Behavioral challenges	*“She will not BUDGE on anything that she has decided needs to be her way. She will have total meltdowns if we try to* *change anything. She has been known to start screaming if we try to change one of her routines.”*
	Slow developmental progress	*“The greatest frustration is [my son’s] lack of progress. We have ABA therapy but he seems to take one step forward and two steps back.”* *“Also, the slow progress is disheartening especially when the child tries so hard. Other people give up too easily- school, therapists, family…”*
	Lack of independence with self-care activities	*“The lack of independent skills even including fully dressing and toileting, needs help with eating, *etc*. most children with DS are able to become somewhat independent in these basic areas. They can speak and interact with others.”*
	Need for constant supervision	*“People don't understand that he is a runner and that (…) I can’t just leave him with anyone (the free respites are nice but there isn't anyone qualified to watch him).”*
	Lack of knowledge from educators and providers	*“Fighting for services/advocating for my son. It debilitates me.”* *“Fighting with the people who are supposed to be helping us. The schools, doctors, therapists, *etc*. have more often than not been working against us or simply no help.”*
Impact on the family unit	Social isolation	*“Trying to do ANYTHING as a family because his social aversions continue to be so significant and disruptive. (…)* *We have never gone to a restaurant, movie theater, or* *public swimming pool as a family. We have never taken a family vacation and either significantly disrupted or been forced to leave every family celebration (weddings, funerals, graduations, *etc*.) we've attempted to attend.”*
	Impact on career, marriage	*“Not having more children; we didn’t think we could handle more…. Career trajectory flatlined. Marriage taxed: not always energy for each other.”*
	Increased empathy	*“I cannot understate the degree to which this has impacted my children in their lack of judgment when faced with the experience of socializing or spending time alongside a same-aged peer with significant differences. They have learned lessons from him my husband and I would have never been able to directly teach and they're far better people already at their ages than I ever was during the same periods of my own life”*
	Worrying about the future	*“The future seems more unclear than that of families with a DS child. There seem to be so many more opportunities for them to plan for. Depending on where a person is on the spectrum the same seems true for autistic people. Now, we feel that he will always live with us and have a full time care giver.”*
How the caregiver experience differs from that of caregivers of individuals with DS alone or ASD alone	Compound disability	*“The DS/ASD confuses him and us. [My son]'s lack of communication and fear of change makes his developmental disfunction exponential.”* *“The "difficulties" we experience as part of her [DS] diagnosis are so insignificant compared to what we're constantly up against with [my son with DS-ASD] that they truly do not factor in to how we handle our daily lives*
	Lack of specialized interventions	*I think they are vastly different than parents of children with only DS. The interventions we use are more focused on the symptoms of our child's autism. However, it seems there is a lot of disagreement in the autism community and we haven't wanted to get involved”*
	Lack of belonging to either community	*“Then when you are at ASD events the other parents think we don't belong because the ASD presents differently in our kids so they think we shouldn't be there”* *“In short, I think our experiences are challenged by multiple facets not facing families with a singular diagnosis. I DO think it's harder. And lonelier. And involves more opportunities for questioning our effectiveness and confidence as parents due to the increased communities in which comparing our son to others with a similar diagnosis leaves our son always coming up short.”*
	Feeling judged by other parents	*“As a mother I’ve repeatedly heard his ASD is just behavior I have promoted by spoiling him or enabling him to do whatever he wanted. It’s my fault he acts the way he does. I should put him in a nursing home & get my life back. When I don’t put him away in a home, those judgmental friends & family walk away from us.”* *“My son looks like a kid with Down syndrome. If that's all anyone knows about him and they're in a position to consider my son as the product of the quality of interventions available to a child with that diagnosis my husband and I both look like negligent and incompetent parents”*

## Discussion

This study is the first to look at perspectives of caregivers with a child with DS-ASD. The use of qualitative coding and theme analysis enabled a detailed and nuanced view of family perspectives. Children with DS-ASD are a relatively understudied patient population, and this study offers valuable insight into their families’ lives.

Most survey respondents noticed that their child was different from others with DS in early childhood, with a mean age of first concern at 3.87 years old. In comparison, multiple studies of children with idiopathic ASD have reported initial parental concern before the age of 2 (Baghdadli et al, 2003; Chamak et al, 2011; Chawarska et al, 2007; De Giacomo & Fombonne, 1998; Goin-Kochel et al., 2006; Howlin & Asgharian, 1999; Zuckerman et al., 2015). This study found an average 4.65-year delay between first noticing symptoms and obtaining a diagnosis. This “age gap” is greater than the one reported in a United States- based survey of caregivers of individuals with idiopathic ASD, which found that the mean delay between the first conversation with a provider and diagnosis of ASD was 2.70 years (Zuckerman et al, 2015). Children with DS-ASD in our study were diagnosed at an average age of 8.52 years, which is several years older than the reported average age at diagnosis of 5–6 years in idiopathic ASD (Howlin & Moore, 1997; Mandell et al, 2010; Siklos & Kerns, 2007). Notably, the “age gap” was larger for individuals who were older at the time the survey was completed, suggesting that there has been some improvement in the recognition and diagnosis of ASD in children with DS over the past three decades. It should be noted that this finding could have been confounded by the wide age range of individuals with DS-ASD at the time that the survey was completed.

The identification and diagnosis of ASD in individuals with DS can be complicated by multiple factors. The core symptoms of ASD (social-communicative impairments and repetitive behaviors and interests) may be less pronounced in DS-ASD than in ASD alone (Godfrey et al, 2019). The overlap of symptoms between the intellectual disability and ASD can cause a diagnostic dilemma for providers (Howlin et al, 2008; Reiss et al, 1982); for example, many children with DS and more severe intellectual disability have stereotyped behaviors (Channell et al, 2015; Kraijer & de Bildt, 2005). ASD-like symptoms, as rated on the Social Responsiveness Scale, are more prevalent in individuals with DS without a co-morbid ASD diagnosis than in the neurotypical population (Channell et al, 2015). Hearing loss, hypotonia, and visual impairment, all of which occur with high incidence in individuals with DS, may also affect language and social development (DiGuiseppi et al, 2010; Rasmussen, 2001). While challenging even for an astute provider to know when ASD-like symptoms indicate an ASD diagnosis, the presence of multiple signs and symptoms, as well as a concern that a child is “different” from other children with DS, should prompt further evaluation by a specialist, rather than being dismissed as attributable to DS or intellectual disability alone. Of note, the characteristics that were identified as differentiating children with DS-ASD from other children with DS only, as articulated by survey respondents, are consistent with scoring algorithm items on the Autism Diagnostic Observation Schedule (ADOS)—the “gold standard” diagnostic tool for diagnosing ASD in the general community (Lord et al, 2000). Individuals with DS-ASD have been shown to have similar ADOS profiles as children with idiopathic ASD (Oxelgren et al., 2019).

Parental reports in the survey correlated well with published findings on DS-ASD, further supporting the notion that parental concerns should not be ignored or dismissed. The most commonly reported symptom was stereotypic behavior (n = 24, 53%), consistent with previously published findings of higher rates of stereotypy on the Aberrant Behavior Checklist in children with DS-ASD compared to children with DS only (Capone et al, 2005); the literature has shown that while many children with DS display stereotypic behavior (Howlin & Moore, 1997), those with DS-ASD engage in more persistent, complex and bizarre stereotypy (Carter et al, 2007). Caregivers commonly reported mood and behavior concerns, which supports previous findings of higher rates of behavior difficulties, hyperactivity, and inappropriate speech in individuals with DS-ASD compared to DS only (Capone et al, 2005; Carter et al, 2007; Molloy et al, 2009). Of note, a recent study found that specific types of maladaptive behaviors, such as self-harm and withdrawal, were associated with higher risk of DS-ASD (Channell et al, 2019). In our survey, parents also frequently reported significant communication difficulties, consistent with findings that individuals with DS-ASD have poorer receptive and expressive language abilities and more inappropriate speech than those with DS alone (Magyar et al., 2012; Molloy et al, 2009).

Early diagnosis is a major determinant of outcomes in patients with ASD, since intensive interventions early in life may lead to better outcomes (Greenspan, 1994; Kraijer & de Bildt, 2005; Webb et al, 2014). A common theme among survey respondents was that their concerns were initially dismissed, contributing to the delay in ASD diagnosis. For many of the participants, the journey to a dual diagnosis involved interactions with multiple providers, educators, and therapists before finding one who was able to recognize the signs of ASD and evaluate them; this highlights a lack of awareness of DS-ASD, and suggests the need for education, especially for primary care providers and educators, who are often the first to hear parental concerns.

Having a child with dual diagnosis has a huge impact on a family, both positive and negative. Thirty eight percent of participants reported feelings of isolation, which parents attributed to two major categories—the inability to leave the home or attend family gatherings due to the child’s behavior or sensory issues and being misunderstood as “bad parents” in public due to their child’s symptoms and behaviors. This is consistent with findings that the “DS advantage” – the notion that parents of individuals with DS experience less stress and greater parenting satisfaction when compared with caregivers of children with other developmental disabilities (Hodapp et al., 2001) – does not hold true when controlling for the child’s adaptive behavior (Corrice & Glidden, 2009). The survey results highlight the value of adequate respite services for children with DS-ASD. Parents relied on building a community with the families of other dually diagnosed children, underscoring the importance of connecting families to online and in-person support groups. Studies have found that social support is a very important factor related to the coping of families of children with ASD and helps reduce their stress levels (Luther et al., 2005).

This study provides insight on how we can enhance care for children with DS-ASD through improved understanding of families’ experiences. However, this study had several limitations. Our sample size was small, with only 47 survey meeting inclusion criteria. The survey solicited very limited demographic data, which affects the generalizability of its findings. Surveys were included based on parent report that their child had been diagnosed with DS-ASD, with no independent verification process; given that diagnosing ASD in genetic syndromes is more challenging (Hepburn & Moody, 2011), future research studies should include additional characterization measures of ASD symptomatology, to increase confidence that findings truly describe the DS-ASD population. We did not have a comparative group of families of children with DS alone or ASD alone; future research should include these comparison groups, who may experience similar challenges with school programming and access to services. While the open-ended format of the survey questions allowed respondents to freely express themselves, it also introduced the potential for bias in the process of grouping responses into categories. Given our reliance on survey data, there may be recall bias in the reporting of initial concerns around symptoms, since parents are more familiar with the diagnostic criteria for ASD after their child has been diagnosed. There may also be negativity bias with patients remembering challenges with accessing diagnostic and intervention services, which may have occurred years prior to survey completion.

Participants in our study may not be representative of parents in the general population, as they needed to read and write in English, be members of the DS-ASD connection (the online support group where participants were recruited) and have access to the internet to complete the survey. The results are subject to significant selection bias, as all respondents were members of a support group who may have sought out an online community to cope with more challenging parenting experiences. In studies focusing on idiopathic ASD, being a non-English speaker was associated with delays in ASD diagnosis (Fountain, King, Bearman, 2011; Valicenti-McDermott et al, 2012), suggesting the possibility that children with DS-ASD who come from non-English speaking families, minority groups, or lower socio-economic statuses may be experiencing even greater delays in diagnosis. We hope that this study will prompt further inquiry into the experiences of caregivers of individuals with DS-ASD, including those whose primary language is not English, with more detailed categorization of participants’ racial/ethnic background, education level, and socio-economic status.

## Conclusion

This study corroborates previous findings that diagnosis of ASD in children with DS is significantly delayed (Howlin et al, 2008; Rasmussen et al, 2001). It also substantiates prior research showing that children dually diagnosed with DS-ASD experience greater challenges with behavior, adaptive functioning, and communication than children with DS alone (Magyar et al, 2012; Molloy et al, 2009). This study underscores the importance of validating parental concerns and promptly connecting patients with signs of ASD with appropriate evaluations and services and emphasizes the need for educating primary care providers and educators on the presentation and needs of individuals with DS-ASD. This study also highlights the need for ongoing research on how children with DS-ASD differ from those with DS alone, and the importance of supporting caregivers by connecting them with meaningful respite and supportive services at home and in the community.
